# Morphological patterns of buccal bone plate in the maxillary esthetic zone: A cross‐sectional study

**DOI:** 10.1002/jper.70071

**Published:** 2026-01-07

**Authors:** Danijel Domic, Diogo Moreira Rodrigues, Emilio Couso‐Queiruga, Eliane Porto Barboza, Mariano Sanz, Christian Ulm, Gustavo Avila‐Ortiz

**Affiliations:** ^1^ Division of Oral Surgery University Clinic of Dentistry, Medical University of Vienna Vienna Austria; ^2^ ETEP (Etiology and Therapy of Periodontal and Peri‐Implant Diseases) Research Group University Complutense of Madrid Madrid Spain; ^3^ Department of Periodontology National Institute of Dental Sciences (INCO 25) Niterói Rio de Janeiro Brazil; ^4^ Department of Oral Surgery and Stomatology University of Bern School of Dental Medicine Bern Switzerland; ^5^ Department of Dental Clinic Fluminense Federal University Niterói Rio de Janeiro Brazil; ^6^ Department of Periodontics Lake Erie College of Osteopathic Medicine (LECOM), School of Dental Medicine Lakewood Ranch Florida USA; ^7^ Department of Periodontology Faculty of Dentistry University of Oslo Oslo Norway; ^8^ Department of Periodontics and Oral Medicine University of Michigan School of Dentistry Ann Arbor Michigan USA

**Keywords:** classification, dental radiography, diagnostic imaging, maxilla, phenotype

## Abstract

**Background:**

This cross‐sectional study aimed to characterize buccal bone plate (BBP) morphological patterns in anterior maxillary teeth and to evaluate their association with other phenotypical and local anatomical characteristics.

**Methods:**

Two examiners conducted a qualitative assessment of BBP morphological patterns using cone‐beam computed tomography scans from healthy adults. Validated cases were employed to determine numerical thresholds for buccal bone thickness (BBT) for each BBP category at different apicocoronal levels. Periodontal phenotypical‐ and anatomical variables, including BBT, gingival thickness (GT), keratinized tissue width (KTW), supracrestal tissue height (STH), distance from the cementoenamel junction to the bone crest (CEJ‐BC), sagittal root position (SRP), and tooth shape (TS), were documented through radiographic, clinical, and photographic evaluations.

**Results:**

Four distinct BBP morphological patterns were identified and categorized as rectangular (39.2%), inverted triangular (23.6%), triangular (14.4%), or hourglass (22.8%). Significant variations in mean BBT values across the 4 BBP patterns were observed at different apicocoronal levels (*p* < 0.001). Associations were found between BBP morphological patterns and GT, SRP, and TS (*p* < 0.001). Multilevel logistic regression analysis showed that BBT, regardless of the vertical level, and GT at 2 mm apical to the gingival margin were key predictors of BBP shape. The inverted triangular pattern was linked to a thicker gingival phenotype and was mostly observed in lateral incisors.

**Conclusion:**

Four BBP patterns were identified, and a novel classification system was devised. Associations between different categories and key phenotypic and anatomical variables, including BBT, GT, SRP, and TS, were observed. This classification can be used to aid diagnostic processes, and in the planning and execution of various treatments in the anterior maxilla, as well as in future research.

**Plain language summary:**

The bone that supports the upper front teeth plays an important role in maintaining oral health and ensuring the success of dental treatments. Traditionally, this bone has been described using simple linear measurements. In the present study, we analyzed the entire bone plate and identified 4 distinct shapes: rectangular, inverted triangular, triangular, and hourglass. These shapes were linked to how thick the bone and gums were at different levels, and to the position and shape of the tooth. For example, the inverted triangular shape was seen more often in lateral incisors and was related to thicker gums. Understanding these patterns can help dentists plan treatments such as periodontal surgery and dental implants, and it also gives researchers a new tool for future studies.

## 1 INTRODUCTION

The buccal bone plate (BBP) is a highly relevant component of the tissues that support and surround the teeth. The features of the BBP may influence treatment planning, clinical decision‐making, and the outcomes of various treatments, including unassisted socket healing following tooth extraction,^1^, ^2^ immediate implant placement (IIP),^3^ alveolar ridge preservation (ARP),^4^ surgical crown exposure,^5^ and orthodontic tooth movement,^6^, ^7^ among others. For example, a buccal bone thickness (BBT) ≥1 mm has been proposed as the minimum threshold required to maintain hard tissue architecture predictably and to support the overlying soft tissue after tooth extraction, thereby reducing the risk of aesthetic complications such as apical mucosal migration after IIP.^8^, ^9^

To date, most studies on this topic have involved the radiographic assessment of BBT using cone‐beam computed tomography (CBCT) to obtain horizontal linear measurements at different apicocoronal levels.^10^, ^11^, ^12^, ^13^ A systematic review and meta‐analysis evaluating BBT in the anterior maxilla reported values ranging from 0.23–1.30 mm at the crestal level, 0.40–1.42 mm at mid‐root, and 0.39–2.13 mm at the apical level.^11^ However, these findings are typically presented as mean values, despite marked intra‐ and interindividual variability.^11^, ^14^ BBP is not a uniform structure, as it exhibits considerable variability across its apicocoronal extent.^15^, ^16^ This consideration raises an important question: Does the standard method of isolated linear BBT measurement adequately capture the complexity of the BBP's anatomical morphology?

Several classifications and assessment systems have been proposed to support clinical decision‐making, most of which focus on post‐extraction alveolus morphology^17^, ^18^, ^19^, ^20^ or root position in the anterior maxilla.^21^ For example, Araujo and coworkers proposed a multifactorial system evaluating the number of bone walls, BBT (thin < 1 mm vs. thick ≥1 mm), root angulation relative to the alveolar envelope, and the width of basal bone.^20^ Sabri and colleagues introduced a 3‐type socket classification that incorporates factors such as gingival recession, soft tissue phenotype, BBT, buccal and interproximal bone loss, apical pathosis, and root angulation.^18^ Similarly, Steigmann and coworkers proposed an alveolar socket classification consisting of 3 types with several subtypes explicitly addressing BBP‐related characteristics.^17^ While these systems do consider BBP features, they primarily focus on BBT or the presence of bony defects (i.e., fenestration or dehiscence). Notably, although 1 recent attempt was made to classify BBP patterns in the mandible of periodontally compromised patients,^22^ no analogous system currently exists for the anterior maxilla.

Therefore, this cross‐sectional study aimed to characterize BBP morphological patterns in anterior maxillary teeth and to evaluate the association between BBP patterns and other phenotypical and local anatomical characteristics.

## 2 MATERIALS AND METHODS

### 2.1 Study design, setting, and ethical approval

This cross‐sectional study was conducted following the Strengthening the Reporting of Observational Studies in Epidemiology (STROBE) guidelines.^23^ Data collection was carried out in the Department of Periodontics, Fluminense Federal University (Brazil), between January 2016 and September 2023. Ethical approval for the study protocol was granted by the Ethics Committee of Fluminense Federal University (CEP/HUAP/UFF #506.300), and the study adhered to the principles of the Declaration of Helsinki.^24^

### 2.2 Study population

**FIGURE 1:**
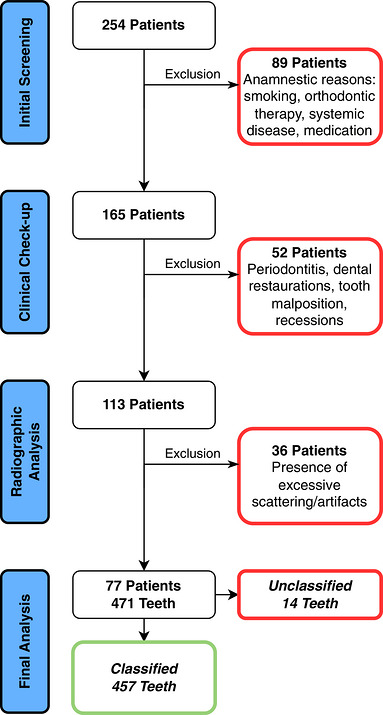
Patient flow diagram according to Strengthening the Reporting of Observational Studies in Epidemiology (STROBE) guidelines.

As previously reported in other studies involving this cohort of patients.^2^, ^14^, ^25^ The inclusion criteria were as follows: (1) adult patients requiring comprehensive dental treatment; (2) age ≥ 18 years; (3) American Society of Anesthesiologists (ASA) physical status I or II; (4) presence of at least 1 maxillary non‐molar tooth bordered by periodontally healthy teeth and with no history of restorative treatment; and (5) availability of a CBCT scan of the region of interest obtained as part of the diagnostic and treatment planning process. Exclusion criteria included: (1) mandibular teeth; (2) maxillary premolars and molars; (3) history of orthodontic or surgical treatment in the anterior maxilla; (4) clinical attachment loss; (5) gingival excess (e.g., pseudopockets, inconsistent gingival margins, excessive gingival display, or gingival enlargement), or abnormal gingival color; (6) mispositioned or crowded teeth; (7) history of dental trauma; (8) teeth with diastema, caries, fractures, resorption, or restorations; (9) uncontrolled diabetes mellitus (HbA1c > 7.0); (10) current smokers; (11) active local or systemic infections; (12) any disease or medication affecting bone or soft tissue metabolism; (13) current chemotherapy or radiotherapy, or a history of radiotherapy to the head and neck region; (14) severe hematologic disorders; (15) pregnant or nursing women; (16) disabilities or barriers that could interfere with understanding, reading, or signing the informed consent. The patient flow diagram is presented in Figure 1.

### 2.3 Data acquisition and assessments

#### 2.3.1 Radiographic

As previously reported,^2^, ^14^, ^25^ a CBCT scan was obtained* following as low as a diagnostically acceptable (ALADA) principle.^26^ Participants were seated with their head and chin stabilized during the exposure. The field of view was approximately 5 cm with a voxel size of 0.11 mm. The exposure settings were standardized at 90 kVp and 4 mAs.

**FIGURE 2:**
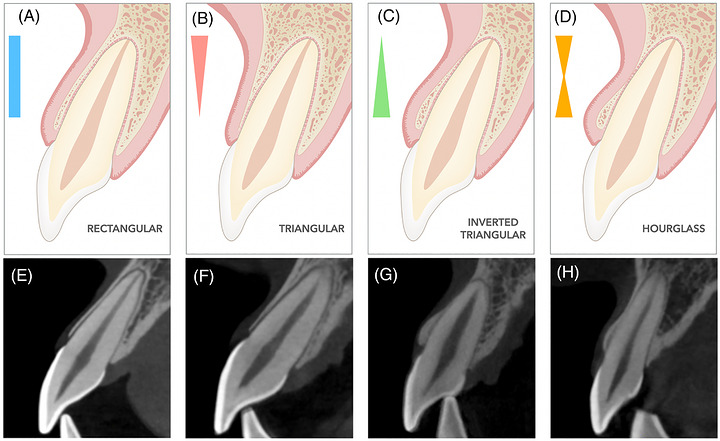
Visual depiction of buccal bone morphological patterns were identified and categorized. Illustrations and radiographic images represent, from left to right, 4 distinct patterns: rectangular (A and E), triangular (B and F), inverted triangular (C and G), and hourglass (D and H).

A 2‐phase methodology was used. First, Digital Imaging and Communications in Medicine (DICOM) files were obtained from the CBCT scans and imported into the software.† A sagittal section at the midline of each randomly selected maxillary tooth (*n* = 102) was generated and visually assessed by a single examiner (D.D.). Based on qualitative observations of BBP characteristics at the crestal, mid‐root, and apical levels, 4 distinct morphological patterns were identified and categorized as rectangular, triangular, inverted triangular, and hourglass, as shown in Figure 2. Subsequently, a second examiner (D.M.R.) was introduced to these patterns and instructed on the qualitative classification criteria for standardization purposes. Both examiners (D.D. and D.M.R.) independently performed blinded, randomized visual assessments of all cross‐sections included in the original sample of this study. Following this, intra‐ and inter‐examiner agreement regarding the BBP shape classification was determined. Only cases with complete (100%) agreement between and within examiners were included in the subsequent quantitative analysis. These consensus‐validated cases represented the final sample and were used to define numerical thresholds for each BBP shape category based on the BBT. The preliminary qualitative classification of the BBP and the final numerical BBP shape classification are presented in Table 1.

**TABLE 1:** Description of each category of the proposed classification for anterior buccal bone plate morphology.

Classification	Definition	Crestal BBT	Mid‐root BBT	Apical BBT	Distinctive feature	Clinical implications^*^
Rectangular	All 3 sections within ± 0.4 mm	Uniform thickness (typically medium‐thick)			Relatively consistent thickness	Favorable, unless thickness is generally ≤0.5 mm
Triangular	BT2‐RA > 1.6x with respect to BT‐MP and BT2‐BC	Thin or dehiscence	Thin‐medium	Thick	Gradual apical thickening	Risk of dehiscence if BBT at the coronal third is ≤0.5 mm
Inverted triangular	BT2‐RA ≤0.6x respective to BT‐MP and BT2‐BC	Thick	Medium‐thin	Thin or fenestration	Gradual apical thinning	Risk of apical fenestration if BBT at the apical third is ≤0.5 mm
Hourglass	BT‐MP ≤0.6x respective to both BT2‐BC and BT2‐RA	Medium‐thick	Thin or fenestration	Medium‐thick	Mid‐root thinning	Risk of mid‐root fenestration

* *Note*: These represent the authors’ opinions and should be confirmed in future studies.

Abbreviations: BBT, buccal bone thickness; BT‐MP, bone thickness at midpoint level; BT2‐BC, bone thickness at 2 mm apical to bone crest; BT2‐RA, buccal thickness at 2 mm coronal from the radiographic apex; mm, millimeter.

**FIGURE 3:**
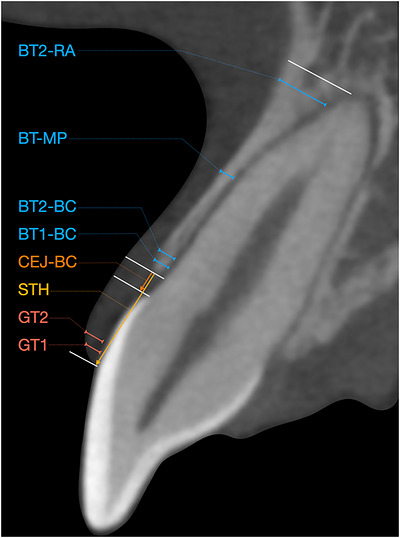
Mid‐sagittal radiographic section demonstrating the methodology applied to make linear measurements of GT1 (gingival thickness at 1 mm apical to the gingival margin); GT2 (gingival thickness at 2 mm apical to the gingival margin); STH (supracrestal tissue height); CEJ‐BC (distance between cementoenamel junction and bone crest); BT1‐BC (buccal bone thickness at 1 mm apical to the buccal crest); BT2‐BC (buccal bone thickness at 2 mm apical to the buccal crest); BT2‐RA (buccal bone thickness at 2 mm coronal from the radiographic apex); and BT‐MP (buccal bone thickness at midway between BT2‐BC and BT2‐RA).

Linear radiographic measurements of anatomical variables of interest were obtained by a single independent examiner (D.M.R) using CBCT scans and the software as mentioned above. To standardize the linear measurements (in mm) of the BBT and gingival thickness (GT), a sagittal section at the midline of each maxillary tooth was selected. Images were evaluated using the maximum zoom that preserved the image quality with standardized contrast and brightness settings on a 32″ flat‐panel monitor with a resolution of 1920 × 1080 pixels. For the assessment of BBT, horizontal lines were drawn at the level of the cemento‐enamel junction (CEJ), 1 and 2 mm apical to the alveolar crest (BT1‐BC and BT2‐BC), 2 mm coronal to the apex (BT2‐RA), and the midpoint between BT2‐BC and BT2‐RA (BT‐MP). Similarly, to assess GT, a horizontal line was drawn at 1 mm (GT1) and 2 mm (GT2) apical to the gingival margin and the level of the cemento‐enamel junction (GT‐CEJ). The distance from the gingival margin to the alveolar bone crest, also known as the supracrestal tissue height (STH),^14^ was assessed by drawing a vertical line. Finally, the sagittal root position (SRP)^21^ and the presence or absence of radiographic bony defects (i.e., fenestration or dehiscence) were recorded. To ensure data quality, the same examiner (D.M.R.) previously conducted linear measurements on 150 maxillary anterior teeth across 25 random patients. An overview of the radiographic parameters is presented in Figure 3.

#### 2.3.2 Clinical

A calibrated examiner (D.M.R.) measured keratinized tissue width (KTW) in 25 randomly selected patients, assessing 150 maxillary anterior teeth over 15 days. KTW was recorded at the mid‐facial aspect of each tooth using a UNC‐15 periodontal probe.‡

#### 2.3.3 Photographic

Standardized intraoral photographs were obtained using a digital camera with a ring flash§ at a 1.5:1 ratio, aperture size of f/32, shutter speed of 1/125 s, ISO 200, and a working distance of 20 cm. Image files had a resolution of 18 megapixels and were saved in Joint Photographic Experts Group (JPEG) format. Participants had the Frankfort plane and the pupillary line parallel to the long axis of the camera lens, and the focal point was centered at the maxillary midline.

All intraoral photographs were analyzed twice within 15 days for calibration purposes using designated software.‖ Crown width (CW) and crown length (CL) of central incisors were measured according to Olsson and Lindhe.^15^ A CW/CL ratio higher than 80% was defined as a square‐shaped tooth and lower than 80% as a triangular‐shaped tooth.^27^

### 2.4 Sample size calculation

A pilot sample size calculation was performed, with each tooth considered as the unit of analysis. While, to our knowledge, this is the first study that assesses the 4 distinct BBP shape categories previously described, the calculation was based on data from previously published similar studies that evaluated the relationship between specific anatomical characteristics and phenotypical features (e.g., GT, BBT, STH, and SRP).^28^, ^29^ The sample size required to compare each periodontal phenotypic parameter across BBP shape categories was estimated by assessing the smallest detectable difference between the group means. A type I error rate of *α* = 0.05 (5%) and a statistical power of the study of 0.80 were predefined. The analysis indicated that 445 teeth would be necessary for a meaningful comparison between STH and BBP patterns.

### 2.5 Statistical analysis

All statistical analyses were performed using statistical software.¶ The Shapiro‐Wilk test was used to assess the normality of the data distribution. Mean values and standard deviations (SD) were calculated for all continuous variables. Categorical variables are presented as frequencies and percentages.

Analysis of variance (ANOVA) and Tukey's post hoc analysis were used for comparisons between the bone shape classes and continuous outcomes. Additionally, a multilevel logistic regression model was used to assess the relationship between BBP shape classes and periodontal phenotypic features, with a random intercept, containing 2 hierarchical levels.# The significance level adopted in the tests of the multilevel logistic regression model was 5%.

## 3 RESULTS

### 3.1 Descriptive analysis of the included sample

A total of 77 patients (51 females and 26 males), with a mean age of 25.6 ± 5.4 years, were included in this study. Regarding race distribution, 60 participants were white, and 17 were black. A total of 471 permanent maxillary anterior teeth were evaluated. Among them, 14 did not meet the predefined numerical criteria for BBP classification; therefore, 457 teeth (151 central incisors, 154 lateral incisors, and 152 canines) were included in the final analysis. Mean mid‐facial KTW was 4.64 ± 1.36 mm. Mean GT1, GT2, and GT‐CEJ were 0.94 ± 0.21 mm, 1.31 ± 0.30 mm, and 1.23 ± 0.33 mm, respectively. Mean STH was 3.26 ± 0.72 mm, while mean CEJ‐BC was 1.76 ± 0.67 mm. Mean BT1‐BC, BT2‐BC, BT‐MP, and BT2‐RA were 0.62 ± 0.25 mm, 0.78 ± 0.42 mm, 0.50 ± 0.40 mm, and 0.67 ± 0.42 mm, respectively. All mean values for periodontal phenotypic variables are displayed in Table 2.

**TABLE 2:** Periodontal phenotypical features dimensions, and their comparison according to buccal bone plate patterns.

Measurements	Overall (*n* = 457) mean ± SD (95% CI)	Rectangular (*n* = 66) mean ± SD (95% CI)	Triangular (*n* = 179) mean ± SD (95% CI)	Inverted triangular (*n* = 108) mean ± SD (95% CI)	Hourglass (*n* = 104) mean ± SD (95% CI)	*p*‐value (ANOVA test)	*p*‐value (Tukey test)
GT1	0.94 ± 0.21 (0.92–0.96)	0.96 ± 0.21 (0.93–0.98)	0.93 ± 0.21 (0.88–0.98)	1.01 ± 0.22 (0.97–1.06)	0.84 ± 0.16 (0.81–0.87)	<0.001^*^	ITS>TS (0.002^*^) ITS>RS (0.019^*^) ITS>HGS (< 0.001^*^)
GT2	1.31 ± 0.30 (1.29–1.34)	1.31 ± 0.30 (1.28–1.37)	1.29 ± 0.30 (1.21–1.38)	1.41 ± 0.29 (1.36–1.47)	1.21 ± 0.30 (1.15–1.27)	<0.001^*^	ITS>TS (< 0.001^*^) ITS>RS (< 0.001^*^) ITS>HGS (< 0.001^*^)
GT‐CEJ	1.23 ± 0.33 (1.20–1.26)	1.23 ± 0.33 (1.17–1.28)	1.28 ± 0.34 (1.20–1.37)	1.26 ± 0.31 (1.20–1.32)	1.16 ± 0.28 (1.11–1.22)	0.002^*^	HGS<TS (0.007^*^) HGS<ITS (0.017^*^)
STH	3.26 ± 0.72 (3.19–3.32)	3.26 ± 0.72 (3.23–3.46)	3.18 ± 0.62 (3.03–3.34)	3.21 ± 0.70 (3.08–3.35)	3.20 ± 0.68 (3.07–3.33)	0.046^*^	p>0.05
CEJ‐BC	1.76 ± 0.67 (1.69–1.82)	1.76 ± 0.67 (1.75–1.95)	1.68 ± 0.58 (1.54–1.82)	1.74 ± 0.62 (1.63–1.87)	1.65 ± 0.75 (1.51–1.88)	0.005^*^	RS>HGS (0.005^*^)
KTW	4.64 ± 1.36 (4.51–4.76)	4.64 ± 1.36 (4.31–4.72)	4.36 ± 1.25 (4.05–4.67)	4.98 ± 1.31 (4.73–5.23)	4.67 ± 0.23 (4.39–4.95)	<0.001^*^	ITS>TS (< 0.001^*^) ITS>RS (< 0.001^*^)
BT1‐BC	0.62 ± 0.25 (0.60–0.65)	0.62 ± 0.25 (0.57–0.63)	0.57 ± 0.24 (0.51–0.63)	0.69 ± 0.26 (0.64–0.74)	0.63 ± 0.26 (0.58–0.68)	<0.001^*^	ITS>TS (< 0.001^*^) ITS>RS (< 0.001^*^)
BT2‐BC	0.78 ± 0.42 (0.74–0.82)	0.78 ± 0.42 (0.59–0.66)	0.60 ± 0.21 (0.55–0.65)	1.21 ± 0.50 (1.12–1.31)	0.72 ± 0.36 (0.65–0.79)	<0.001^*^	ITS>TS (< 0.001^*^) ITS>RS (< 0.001^*^) ITS>HGS (< 0.001^*^)
BT‐MP	0.50 ± 0.40 (0.46–0.54)	0.50 ± 0.40 (0.49–0.57)	0.62 ± 0.30 (0.54–0.69)	0.71 ± 0.57 (0.60–0.82)	0.17 ± 0.15 (0.12–0.18)	<0.001^*^	HGS<ITS (< 0.001^*^) HGS<RS (< 0.001^*^) HGS<TS (< 0.001^*^)
BT2–RA	0.67 ± 0.42 (0.63–0.71)	0.67 ± 0.42 (0.50–0.63)	1.28 ± 0.45 (1.17–1.39)	0.40 ± 0.27 (0.35–0.45)	0.69 ± 0.41 (0.61–0.77)	<0.001^*^	TS>ITS (<0.001^*^) TS>RS (<0.001^*^) TS>HGS (<0.001^*^)

Abbreviations: ANOVA, analysis of variance; BC, bone thickness at 1 mm apical to bone crest; BT1‐BT2‐BC, bone thickness at 2 mm apical to bone crest; BT‐MP, bone thickness at midpoint level; BT2‐RA, bone thickness at 2 mm coronal from radiographic apex; CEJ‐BC, distance between cementoenamel junction and bone crest; CI, confidence interval; GT1, gingival thickness at 1 mm apical to gingival margin; GT2, gingival thickness at 2 mm apical to gingival margin; GT‐CEJ, gingival thickness at the cementoenamel junction; HGS, hourglass shape; ITS, inverted triangular shape; KTW, keratinized tissue width; *n*, number; RS, rectangular shape; SD, standard deviation; STH, supracrestal tissue height; TS, triangular shape.

* Indicates statistical significance.

Most sites were categorized as SRP class I (65.9%), followed by class IV (25.6%), class II (8.1%), and class III (0.4%). Finally, regarding CW/CL, 41% and 59% of the central incisors were categorized as having triangular and square crown shapes, respectively.

### 3.2 Intra‐examiner reliability

An intra‐examiner kappa value of ≥0.85 was achieved regarding linear measurements of periodontal phenotypical features. For the clinical and photographic assessments, an intra‐class correlation of > 0.90 was observed.

### 3.3 Novel buccal bone plate classification according to demographic and phenotypic features

The following distribution for different BBP shapes was found:

- Rectangular, *n* = 179 (39.2%)
- Inverted triangular, *n* = 108 (23.6%)
- Triangular, *n* = 66 (14.5%)
- Hourglass, *n* = 104 (22.7%)

Of the 179 teeth classified as having a rectangular BBP shape, 83 were central incisors, 42 were lateral incisors, and 54 were canines. Among the 108 teeth with an inverted triangular shape, the majority were lateral incisors (*n* = 52), followed by 34 canines and 22 central incisors. The triangular BBP shape was mainly observed in central incisors (*n* = 41), with fewer cases in the canines (*n* = 20) and lateral incisors (*n* = 5). The hourglass shape was found in lateral incisors (*n* = 55) and canines (*n* = 44) and rarely observed in central incisors (*n* = 5). The detailed frequency distribution of the BBP shapes by tooth type is presented in Table 3.

**TABLE 3:** Distribution of sites according to buccal bone plate morphological patterns.

Pattern	Central incisors Number (%)	Lateral incisors Number (%)	Canines Number (%)
Rectangular	83 (18.2%)	42 (9.2%)	54 (11.8%)
Inverted triangular	22 (4.8%)	52 (11.4%)	34 (7.4%)
Triangular	41 (9.0%)	5 (1.1%)	20 (4.4%)
Hourglass	5 (1.1%)	55 (12.0%)	44 (9.6%)

#### 3.3.1 Association between BBP shapes and categorical variables

No significant association was found between BBP shape and sex (*X* ^2^, *p* = 0.098). In contrast, significant associations were found between BBP shape and tooth type (*X* ^2^, *p* < 0.001), SRP classification (*X* ^2^, *p* < 0.001), and gingival phenotype. Specifically, significant associations were found using thresholds of 1.0 mm and 1.2 mm at GT1 (*X* ^2^, *p* < 0.001), and 1.2 mm at GT2 (*X* ^2^, *p* < 0.001) to differentiate thin versus thick gingival phenotypes.

#### 3.3.2 Comparison between BBP shapes and continuous variables

Statistically significant differences were observed in mean BBT values measured at BT2‐BC, BT‐MP, and BT2‐RA across the 4 BBP shapes: BT2‐BC (*p* < 0.001), BT‐MP (*p* < 0.001), and BT2‐RA (*p* < 0.001). In addition, significant differences were seen in mean values of KTW (*p* = 0.009), GT1 (*p* < 0.001), GT2 (*p* < 0.001), and BT1‐BC (*p* = 0.011) among the different BBP pattern groups. Conversely, no statistically significant differences were found between groups regarding mean values of GT‐CEJ (*p* = 0.055), STH (*p* = 0.263), or CEJ‐BC (*p* = 0.093).

Post‐hoc Tukey analysis revealed that BT2‐BC was significantly lower in the rectangular group than in the inverted triangular group (mean difference: −0.592 mm; *p* < 0.001) and significantly higher in the inverted triangular group than in both the triangular (0.617 mm; *p* < 0.001) and hourglass groups (0.490 mm; *p* < 0.001). At the BT‐MP level, sites with rectangular shapes exhibited significantly lower BBT than the inverted triangular group (−0.179 mm; *p* < 0.01), but significantly higher BBT than the hourglass group (0.377 mm; *p* < 0.01). Additionally, both the inverted triangular and triangular groups had significantly greater BT‐MP than the hourglass group (0.556 mm and 0.466 mm, respectively; *p* < 0.001). At the BT2‐RA level, the rectangular group demonstrated significantly higher BT2‐RA than the inverted triangular group (0.191 mm; *p* < 0.001), but significantly lower than the triangular group (−0.685 mm; *p* < 0.001). The inverted triangular group also showed a significantly lower BT2‐RA than both the triangular (−0.876 mm; *p* < 0.001) and hourglass groups (−0.290 mm; *p* < 0.001). Finally, the triangular group exhibited a significantly greater BT2‐RA than the hourglass group (0.586 mm; *p* < 0.001). The mean values of the phenotypical dimensions stratified by BBP patterns are depicted in Table 2.

#### 3.3.3 Multivariate model assessment related to novel buccal bone morphology

Multilevel logistic regression analysis was conducted to evaluate the relationship between phenotypic features and the 4 different BBP patterns, using the rectangular BBP shape as the reference group. When comparing the inverted triangular shape to the rectangular shape, BT2‐BC (estimate = 32.65, *p* < 0.001), BT‐MP (estimate = 6.81, *p* < 0.001), and BT2‐RA (estimate = –39.02, *p* < 0.001) were significant predictors. GT2 was also a significant predictor (estimate = 3.11, *p* = 0.032). For the triangular shape versus the rectangular shape comparison, BT2‐BC (estimate = –7.94, *p* = 0.002), BT‐MP (estimate = –9.37, *p* < 0.001), BT2‐RA (estimate = 21.34, *p* < 0.001), and GT2 (estimate = 4.54, *p* = 0.017) were significant predictors. In the comparison between the hourglass shape and the rectangular shape, the model identified BT2‐BC (estimate = 21.37, *p* < 0.001), BT2‐RA (estimate = 23.87, *p* < 0.001), and BT‐MP as strong negative predictors (estimate = –70.81, *p* < 0.001). Other evaluated parameters, including GT1, GT‐CEJ, STH, CEJ‐BC, and BT1‐BC, did not show statistical significance in most comparisons. Table 4 presents the results of the multivariate model.

**TABLE 4:** Multivariate model analysis.

Model coefficients—Classification						
Classification	Predictor	Estimate	SE	*Z*	*p*	Odds ratio
ITS ‐ RS	Intercept	−11.15155	3.242	−3.43961	<0.001^*^	1.44e0‐5
	BT2‐BC	32.65018	5.534	5.90024	<0.001^*^	1.51e+14
	BT‐MP	6.80958	1.989	3.42398	<0.001^*^	906.4911
	BT2‐RA	−39.02307	7.008	−5.56830	<0.001^*^	1.13e‐17
	GT1	−0.57435	2.564	−0.22397	0.823	0.5631
	GT2	3.11070	1.450	2.14497	0.032^*^	22.4367
	GT‐CEJ	−2.02944	1.835	−1.10579	0.269	0.1314
	STH	−0.39284	0.540	−0.72729	0.467	0.6751
	CEJ‐BC	−0.62279	0.672	−0.92715	0.354	0.5364
	BT1‐BC	−0.92870	1.752	−0.53003	0.596	0.3951
TS ‐ RS	Intercept	−15.41753	3.291	−4.68445	<0.001^*^	2.01e0‐7
	BT2‐BC	−7.93941	2.517	−3.15384	0.002^*^	3.56e0‐4
	BT‐MP	−9.36930	2.534	−3.69724	<0.001^*^	8.53e0‐5
	BT2‐RA	21.34307	3.173	6.72752	<0.001^*^	1.86e0+9
	GT1	−0.63060	2.001	−0.31512	0.753	0.5323
	GT2	4.54359	1.903	2.38710	0.017^*^	94.0276
	GT‐CEJ	−0.45877	1.573	−0.29169	0.771	0.6321
	STH	0.36806	0.824	0.44680	0.655	1.4449
	CEJ‐BC	0.07972	0.920	0.08665	0.931	1.0830
	BT1‐BC	0.87902	1.336	0.65789	0.511	2.4085
HGS ‐ RS	Intercept	−0.22768	3.718	−0.06124	0.951	0.7964
	BT2‐BC	21.36954	3.326	6.42479	<0.001^*^	1.91e0+9
	BT‐MP	−70.81129	9.526	−7.43342	<0.001^*^	1.77e‐31
	BT2‐RA	23.87170	3.369	7.08674	<0.001^*^	2.33e+10
	GT1	−2.65271	2.940	−0.90242	0.367	0.0705
	GT2	−1.44142	1.845	−0.78130	0.435	0.2366
	GT‐CEJ	−0.00601	1.904	−0.00316	0.997	0.9940
	STH	−0.19851	0.843	−0.23556	0.814	0.8200
	CEJ‐BC	−0.36816	0.862	−0.42686	0.669	0.6920
	BT1‐BC	0.14720	1.773	0.08304	0.934	1.1586

Abbreviations: BT1‐BC, bone thickness 1 mm from bone crest; BT2‐BC, bone thickness 2 mm from bone crest; BT‐MP, bone thickness at midpoint level; BT2‐RA, bone thickness at 2 mm coronal from radiographic apex; CEJ‐BC, distance from cementoenamel junction and bone crest; GT1, gingival thickness 1 mm from gingival margin; GT2, gingival thickness 2 mm from gingival margin; GT‐CEJ, gingival thickness at the cementoenamel junction; HGS, hourglass shape; ITS, inverted triangular shape; KTW, keratinized tissue width; RS, rectangular shape; STH, supracrestal tissue height; TS, triangular shape.

^*^ Indicate statistical significance.

### 3.4 Unclassified buccal bone plate morphology

The proposed categories are mutually exclusive, ensuring that each tooth meeting at least 1 shape criterion is assigned to a single distinctive category. However, a small number of cases (*n* = 14, 3.4%) did not meet the predefined criteria (Table 1) due to abnormal anatomical variations or due to borderline characteristics and were, therefore, categorized as “unclassified.” These cases were excluded from further statistical analyses.

## 4 DISCUSSION

To date, this is the first study to introduce and validate a classification system for different BBP patterns, primarily using midsagittal CBCT measurements of maxillary anterior teeth with minimal or no signs of gingival inflammation and attachment loss. Four morphologically distinct BBP shapes were defined and classified as rectangular, triangular, inverted triangular, and hourglass. Each shape exhibited distinct morphometric profiles, with statistically significant differences in BBT across the 3 vertical levels (i.e., BT2‐BC, BT‐MP, and BT2‐RA). BBP shapes also correlated significantly with phenotypic and anatomical features, including GT, KTW, SRP, and tooth type. Importantly, multilevel logistic regression identified BBT at 3 levels (i.e., BT2‐BC, BT‐MP, and BT2‐RA) and GT2 as significant predictors of BBP shape, reinforcing the plausibility of the proposed classification and offering a foundation for future research and clinical/diagnostic application.

The rectangular shape, characterized by a relatively uniform BBT across all levels (± 0.4 mm), was the most prevalent (39.2%). The inverted triangular shape (23.6%), defined by crestal thickening and apical thinning (BT2‐RA ≤0.6× for BT2‐BC/BT‐MP), was primarily observed in lateral incisors and was associated with a thick gingival phenotype, which we hypothesize may be favorable, for example, when IIP is performed. The triangular shape (14.5%) exhibited apical thickening (BT2‐RA ≥1.6× for BT2‐BC/BT‐MP) and thinner crestal bone, potentially indicating an increased risk for dehiscence following unassisted extraction socket healing. Finally, the hourglass shape (22.7%) showed significant mid‐root thinning (BT‐MP ≤0.6× for BT2‐BC and BT2‐RA). This class was strongly associated with a thin gingival phenotype and SRP class I, suggesting a higher likelihood of soft tissue dehiscence and/or mid‐root fenestration during surgical procedures and orthodontic tooth movement.

Several variables have been identified as prerequisites for successful IIP and ARP, particularly in the esthetic zone. These include BBT ≥1 mm, thick GT (≥1 mm), KTW ≥2 mm, SRP class I or II, and a minimum of 2 mm of apical bone for implant primary stability.^2^, ^8^, ^30^, ^31^ In the proposed classification, the inverted triangular shape fulfilled many of these anatomical requirements, especially among lateral incisors. However, this shape displayed significant apical thinning (mean BT2‐RA = 0.40 ± 0.27 mm), which could increase the risk of apical fenestration. Similar findings were reported by Zhang and colleagues, who described a “Type II” alveolus exhibiting comparable apical characteristics and lateral incisor predominance.^19^ While apical bone thinning may pose a challenge in these sites, the possibility of buccal bone perforation could be compensated for with the use of tapered implants presenting a narrower diameter in the apical segment. Interestingly, Couso–Queiruga and coworkers reported a 97% feasibility rate for IIP in maxillary lateral incisors when respecting incisal‐edge emergence protocols.^3^ Additionally, maintaining a horizontal gap of ≥2 mm between the implant and the inner part of the BBP, along with ARP via socket filling of the horizontal gap with a biomaterial presenting low biodegradability, could mitigate potential functional and esthetic complications.^32^ Although our study was cross‐sectional and limited to anatomical observations, all BBP shapes, except the triangular shape, may be favorable for IIP based on their anatomical characteristics, though clinical trials are needed to confirm this hypothesis. And even in sites with unfavorable anatomical features, adopting specific therapeutic strategies (e.g., simultaneous soft tissue augmentation with an autogenous connective tissue graft and careful transmucosal abutment design) can increase the predictability of IIP‐based approaches. Nonetheless, further evidence on remodeling patterns across different BBP morphologies is needed to substantiate this assumption.

Previous studies have predominantly reported mean BBT values at one or more levels, but to our knowledge, the morphological variability of the BBP has not been comprehensively assessed in other investigations.^10^, ^11^, ^12^, ^13^ Our study revealed significant differences among the 4 BBP patterns. Importantly, the overall mean BBT at BT2‐BC did not reach the 1 mm desired threshold often cited as necessary to preserve most of the pre‐existing hard and soft tissue architecture after unassisted extraction socket healing. In general, the overall mean values observed in this study are slightly higher than those reported in a previous study published using a similar methodology.^33^ In that study, the maximum BBT was measured within the most coronal and apical 3 mm of the BBP, and at a mid‐point between these two.  Interestingly, Younes and coworkers reported similar BT2‐BC values for lateral incisors compared with the present study. Furthermore, they reported that BBT ranged between 1 and 2 mm at BT2‐BC in 48% of lateral incisors, and in 8% was ≥2 mm, which was higher than in the central incisors and canines.^34^ The greatest GT was observed 3 mm apical to the gingival margin, which aligns with our overall findings at the GT2 level.^34^

Other periodontal parameters, such as STH, KTW, CEJ‐BC, and GT at the level of the bone crest, have been previously investigated and correlated with BBT.^14^, ^25^ A negative correlation between CEJ‐BC and BT1‐BC, GT, and KTW has been reported.^14^ Hence, in general, the shorter the CEJ‐BC distance, the higher the BBT, GT, and KTW. Interestingly, in our study, no significant difference was found in the mean values of CEJ‐BC among the 4 different BBP shapes (*p* = 0.093). Another clinically relevant parameter, gingival stippling, has been associated with higher BT1‐BC, GT, and KTW.^25^ In our study, this parameter was not investigated; however, it may be plausible that gingival stippling, which is primarily dictated by specific features of the gingival epithelium,^35^ could be associated with specific BBP patterns. Thus, this assessment should be included in future studies. In the present study, no differences in the distribution or frequencies of the proposed classification with respect to sex were found.

Measurements were taken at 3 vertical levels to accurately capture the entire morphology of the BBP while reducing measurement error and enhancing clinical relevance. It was assumed that, in the coronal third, the highest BBT usually appears about 2 mm apical to the crest, so our measurement reference point was set at BT2‐BC. This method was then applied in the apical area, with BT‐MP representing the exact midpoint between crestal and apical measurements. For the GT measurements, 2 thresholds were used: ≥1.0 mm at GT1 and ≥1.2 mm at GT2, based on previous findings by Rodrigues and colleagues.^36^ It should be noted that selecting different measurement points for the BBP and/or using alternative threshold values for GT might influence the frequency distribution of the 4 BBP shapes and their correlations with other parameters.

Although high methodological standards were followed, this study had several limitations. The recruited subjects represent a young, systemically healthy Brazilian population, which limits the generalizability to other populations or ethnicities. Moreover, our study population is not necessarily representative of patients with failing dentition who typically seek tooth replacement therapy with dental implants. However, young and healthy individuals may still be candidates for immediate implant placement in certain scenarios or for procedures such as esthetic and/or functional crown lengthening. Second, despite the high resolution of CBCT used herein, assessing the BBP contour, obtaining linear measurements, and distinguishing soft tissue boundaries were challenging in some sites, which may have influenced the reliability of the final linear measurements and soft tissue phenotype assessments, particularly in teeth with thin bone and gingival phenotypes.^13^, ^37^ Moreover, BBP was observed and measured only in the mid‐sagittal section; however, the BBT and BBP shape may vary further mesially or distally from the center. Third, the current classification focuses on the anterior maxillary teeth only and may not apply to the posterior regions or mandible. Although the classification system demonstrated strong discriminatory power and mutual exclusivity, a small subset of the teeth (3.4%) could not be classified. These unclassified cases often reflected borderline or mixed morphologies/anatomical variations that did not meet the strict threshold criteria. For example, 1 case showed unusual thickening at the BT‐MP level (2.50 mm) but had decreased BBT toward BT2‐BC (1.57 mm) and BT2‐RA (2.16 mm) levels. In other borderline cases, the majority of BBP showed either a consistently high BBT at all 3 measured levels (e.g., all BBT measurements > 1 mm) or an overall medium thickness with a favorable BBP shape (e.g., closest to inverted triangular or parallel shape). Interestingly, among the unclassified cases, not a single BBP showed BBT < 1 mm at all 3 measured levels, suggesting that these borderline phenotypes may still represent a favorable clinical situation for IIP or ARP. Future refinements, such as adding transitional categories, adjusting thresholds to identify anatomical outliers, or incorporating machine‐learning‐based probabilistic models, could improve and expand this classification and enhance its clinical utility.

## 5 CONCLUSION

Four distinct BBP patterns were identified, which led to the proposal of a novel classification system that can serve as a diagnostic tool to guide planning and execution of various treatments in the anterior maxilla. While specific categories within this system were associated with key phenotypical and anatomical factors in this study, further research is needed to validate the system across different populations and to evaluate its ability to predict outcomes of diverse therapeutic approaches. Integrating this classification into digital workflows and AI‐powered diagnostics could further enhance clinical decision‐making and improve treatment outcomes in the maxillary esthetic zone.

## AUTHOR CONTRIBUTIONS

**Danijel Domic and Gustavo Avila‐Ortiz**: Conceived and designed the study. **Danijel Domic and Diogo Moreira Rodrigues**: Contributed to data acquisition and analysis. **Emilio Couso‐Queiruga and Gustavo Avila‐Ortiz**: Contributed to data interpretation. **Danijel Domic and Emilio Couso‐Queiruga**: Led the writing. **Diogo Moreira Rodrigues, Eliane Porto Barboza, Mariano Sanz, Christian Ulm, and Gustavo Avila‐Ortiz**: Critically revised the manuscript. All authors gave their final approval and agreed to be accountable for all aspects of this scientific work.

## CONFLICT OF INTEREST STATEMENT

The authors have no conflicts of interest to report pertaining to the conduction of this study.

## Data Availability

The dataset generated during the conduction of the present study is available from the corresponding author upon reasonable request.

## Footnotes

* Prexion 3D Elite, Prexion Inc., San Mateo, CA, USA.

† Prexion 3D Viewer Software, Prexion Inc., San Mateo, CA, USA.

‡ PCP UNC15, Hu‐Friedy, Chicago, IL, USA.

§ Canon 30D SLR, Canon, Tokyo, Japan.

‖ ImageJ, National Institutes of Health, Bethesda, MD, USA.

¶ SPSS version 30.0, IBM Corp., Chicago, IL, USA.

# STATA 11.0, StataCorp LLC, College Station, TX, USA.
